# Nickel Plating

**Published:** 1874-04

**Authors:** 


### NICKEL PLATING.

Within a few years nickel plating has come into such general use that we receive frequent queries in reference to the details of the operation. In answer to these we may say, in the first place, that the whole process is the most difficult that is used in the arts, just in proportion as the results are the most valuable. It is far easier to gild, plate or copper an article than to nickel it, and amateurs who have succeeded so well with silver and copper are likely to be discouraged by the difficulties to be overcome in depositing nickel. Those who have succeeded, after spending much time and money, in making some important improvement, generally keep their method a secret or cover it with patents.

The following description of the usual process is given by Prof. Sharples, in a recent number of the Boston *Journal of Chemistry:*

"The double sulphate of nickel and ammonium, which is the salt that is generally used, may now be had in commerce almost pure. It is manufactured on a large scale by Joseph Wharton, of Camden, N. J., who controls the nickel market in this country. Cast nickel plates for anodes may be obtained from the same source. The anodes should considerably exceed in size the articles to be covered with nickel. Any common form of battery may be used. Three Daniell's or Smee's cells, or two Bunsen's connected for intensity, will be found to be sufficient. The battery power must not be too strong, or the deposited nickel will be black. A strong solution of the sulphate is made and placed in any suitable vessel; a glazed stone-ware pot answers very well if the articles to be covered are small. Across the top of this are placed two heavy copper wires, to one of which the articles to be covered are suspended, to the other the anode. The wire leading to the zinc of the battery must then be connected with the wire from which the articles are suspended, the other wire being connected with the anode.

In order to prepare the articles for coating, they must be well cleaned by first scrubbing them with caustic soda or potash, to remove any grease, and then dipping them for an instant in aqua regia water, taking care that the hand does not come in contact with any part of them. This is accomplished by fastening a flexible copper wire around them and handling them by means of it. The wire serves afterward to suspend them in the bath.

If the articles are made of iron or steel, they must be first covered with a thin coat of copper. This is best done by the cyanide bath, which is prepared by dissolving precipitated oxide of copper in cyanide of potassium. A copper plate is used as an anode. After they are removed from the copper bath, they must be washed quickly with water and placed in the nickel bath; if allowed to dry or become tarnished the nickel will not adhere. Great care must be used through the whole process to keep all grease, dust or other dirt from the articles to be covered, or else the result will be unsatisfactory."

Persons unaccustomed to the use of a battery will not succeed, at first, in getting a constant current of the required strength. Especial care should be given to the amalgamation of the zinc plates, which, on being placed in dilute sulphuric acid, should not "sing" or give off hydrogen gas. If the mercury refuses to adhere to the zinc, the latter may be placed in acid for a few minutes, and the mercury again applied.—*Journal of Applied Chemistry.*

### ROOM ENOUGH AT THE TOP.

"Don't be half of anything," says a modern writer. Sounder advice was never given in more concise language. The bane of many a man's life is that he is never more than half of anything. Sometimes this is the result of trying to be and do too many things at once, but oftener of not exerting himself sufficiently to be a whole something. Very often men are capable of doing one thing well. By that we mean as well as it can be done—not passably, or fairly, but thoroughly, completely and as it onght to be done. It is true there have been exceptions, but those only prove the rule. The men who have made their mark in the world are those who have done one thing, and that thing well. Agassiz was a recent and marked instance of this kind of men. Benjamin Thompson, Count von Rumford was a sample of the other kind—a *rara avis*, to be sure —who did many things, and did them all well; but he wrote himself out, and at the early age of fifty-two he passed out of sight, though he lived some years after, a rather disreputable sort of life, which cast a shade over his former achievements. Agassiz, on the contrary, died in the hight of his fame, leaving a name for future ages as one who did his work well.

The injunction to "do nothing by halves" will apply to all classes of men, whether they belong to the learned profession or are engaged in business or the mechanic arts. We think it was Daniel Webster who said there was plenty room at the top. There is always a surplus of men, and women too, who are competent to fill the middle and lower positions, but a scarcity of those who can take the higher ones. The great want of the world is thoroughness, and the mistake which men make, when starting in life, is in not striving for it. There are multitudes of men and women who all their lives suffer themselves to be underlings, and complain that they are such, who might lift themselves out of such positions if they really and earnestly desired to do so. The trouble is, they want somebody else to do the lifting.

So we revert to our text. Don't be half of anything. Young man, young woman—you who are just starting out in life—remember this injunction. Be a whole something. Put your whole soul into whatever you undertake. Don't let your hands do one thing, and your head another. Don't waste the strength of your body in daily toil for a bare living, and then throw away all your brain power in foolish, senseless and unprofitable things. When head and hands work together in common partnership anything in reason can be achieved. When they work on separate jobs the chances are in favor of a terrible failure. The history of the world is full of examples to prove the truth of our assertion, and we need not cite any of them. But if you prefer to divide your forces—to give your hands to labor and your brain to idleness or mere pleasure—do not complain if you are compelled to remain an underling all your lives, nor envy your fellow men and women who achieve the success which you wish, but do not strive for. Again, we say, don't be half of anything. Aim to be a whole man or a whole woman; be determined to go up higher than you are, always remembering that there is room enough at the top.—*Lynn Reporter.*

